# Supplementary material for: Vitamin B12 Ameliorates Pesticide-Induced Sociability Impairment in Zebrafish (Danio rerio): A Prospective Controlled Intervention Study
Source: Animals (Basel). 2024 Jan 26;14(3):405. doi: 10.3390/ani14030405 (PMC10854844; doi:10.3390/ani14030405)
Supplement: Supplementary file 1 [file animals-14-00405-s001.zip › animals-2791762-supplementary.pdf]

Table S1 – Superoxide dismutase results

NR. SAMPLE	UNTREATED GROUP			VITAMIN GROUP			PESTICIDE GROUP			MIXTURE GROUP		
1	3.546	3.553	3.551	46.227	46.226	46.219	9.273	9.272	9.269	1.556	1.553	1.55
2	9.953	9.956	9.952	39.776	39.771	39.764	4.25	4.248	4.242	5.147	5.143	5.138
3	3.859	3.857	3.863	32.458	32.457	32.452	9.629	9.628	9.624	2.992	2.988	2.984
4	6.592	6.607	6.606	45.91	45.9	45.87	9.604	9.603	9.601	2.663	2.66	2.657
5	7.604	7.602	7.601	56.706	56.702	56.701	6.376	6.373	6.369	4.814	4.811	4.809
6	8.532	8.527	8.524	36.564	36.562	36.56	7.604	7.603	7.6	3.511	3.509	3.505
7	9.305	9.303	9.299	26.635	26.634	26.631	4.993	4.992	4.989	1.943	1.94	1.937
8	4.575	4.575	4.558	53.904	53.903	53.9	3.881	3.88	3.876	2.748	2.744	2.742
9	3.94	3.936	3.93	41.638	41.636	41.632	5.972	5.97	5.965	3.6	3.594	3.593
10	4.782	4.781	4.79	38.518	38.516	38.512	4.6311	4.629	4.627	2.705	2.704	2.7
11	6.61	6.609	6.603	30.51	30.506	30.501	8.808	8.8055	8.801	3.681	3.679	3.675
12	8.593	8.591	8.584	47.693	47.692	47.69	6.845	6.84	6.838	1.832	1.831	1.824
13	6.942	6.937	6.935	53.831	53.829	53.826	7.799	7.794	7.791	2.844	2.843	2.841
14	8.725	8.719	8.713	51.549	51.546	51.543	6.83	6.829	6.827	5.742	5.741	5.7415
15	7.807	7.803	7.799	43.11	43.108	43.105	4.921	4.919	4.915	4.449	4.446	4.445
**MEAN**	**6.758**	**6.757**	**6.754**	**43.002**	**42.999**	**42.994**	**6.761**	**6.759**	**6.756**	**3.348**	**3.346**	**3.343**

Table S2 – Glutathione peroxidase results

NR. SAMPLE	UNTREATED GROUP			VITAMIN GROUP			PESTICIDE GROUP			MIXTURE GROUP		
1	0.237	0.236	0.234	1.893	1.892	1.889	0.123	0.122	0.117	0.115	0.114	0.11
2	0.131	0.13	0.129	0.484	0.483	0.48	0.151	0.15	0.149	0.111	0.11	0.109
3	0.395	0.392	0.391	0.797	0.795	0.792	0.106	0.104	0.102	0.131	0.129	0.127
4	0.14	0.139	0.132	0.45	0.44	0.41	1.027	1.024	1.02	0.204	0.202	0.201
5	0.612	0.611	0.609	2.606	2.605	2.602	0.183	0.182	0.178	0.108	0.106	0.102
6	0.562	0.56	0.558	2.02	2.016	2.015	0.212	0.21	0.209	0.119	0.118	0.113
7	0.354	0.353	0.351	0.821	0.819	0.816	0.174	0.173	0.171	0.174	0.173	0.17
8	1.044	1.043	1.041	1.715	1.714	1.709	0.124	0.122	0.121	0.725	0.722	0.72
9	0.255	0.252	0.251	1.31	1.309	1.304	0.171	0.17	0.168	0.192	0.191	0.187
10	0.196	0.195	0.189	2.461	2.46	2.458	0.094	0.091	0.088	0.163	0.162	0.16
11	0.263	0.262	0.26	0.903	0.901	0.899	0.105	0.102	0.101	0.131	0.13	0.126
12	1.045	1.044	1.043	2.824	2.822	2.82	0.081	0.08	0.076	0.121	0.12	0.119
13	0.236	0.235	0.229	1.66	1.65	1.62	0.135	0.132	0.129	0.116	0.114	0.11
14	1.031	1.029	1.028	3.517	3.514	3.511	0.104	0.102	0.099	0.103	0.102	0.1
15	0.412	0.411	0.41	1.824	1.821	1.82	0.113	0.111	0.109	0.102	0.1	0.099
**MEAN**	**0.460**	**0.459**	**0.457**	**1.685**	**1.682**	**1.676**	**0.193**	**0.191**	**0.189**	**0.174**	**0.172**	**0.170**

Table S3 – Malondialdehyde results

NR. SAMPLE	UNTREATED GROUP			VITAMIN GROUP			PESTICIDE GROUP			MIXTURE GROUP		
1	16.371	16.37	16.365	78.206	78.205	78.201	75.328	75.325	75.322	10.434	10.433	10.429
2	50.767	50.766	50.761	51.433	51.43	51.429	27.559	27.557	27.556	26.041	26.039	26.033
3	20.693	20.692	20.688	67.904	67.902	67.901	34.235	34.232	34.23	25.49	25.489	25.482
4	49.281	49.278	49.274	49.941	49.94	49.938	28.606	28.604	28.6	16.453	16.452	16.449
5	24.084	24.08	24.076	64.876	64.873	64.869	39.411	39.409	39.405	13.25	13.247	13.246
6	19.576	19.574	19.57	49.511	49.509	49.505	68.306	68.305	68.299	21.533	21.532	21.53
7	36.812	36.811	36.808	65.764	65.763	65.761	56.915	56.914	56.912	15.431	15.429	15.428
8	26.709	26.707	26.701	47.905	47.902	47.901	49.626	49.623	49.62	17.6	17.599	17.598
9	29.762	29.761	29.759	68.607	68.606	68.604	79.922	79.921	79.919	19.704	19.703	19.7
10	38.981	38.978	38.975	59.872	59.869	59.865	39.508	39.507	39.502	22.315	22.312	22.311
11	43.555	43.552	43.549	76.023	76.021	76.017	81.312	81.311	81.308	16.711	16.71	16.704
12	36.186	36.183	36.179	69.804	69.803	69.802	53.413	53.412	53.405	17.849	17.847	17.844
13	28.394	28.391	28.388	58.314	58.311	58.309	41.593	41.59	41.588	18.806	18.805	18.803
14	22.747	22.746	22.741	59.8	59.799	59.795	35.923	35.922	36	21.308	21.307	21.303
15	44.594	44.593	44.59	85.42	85.418	85.417	59.845	59.844	59.843	10.664	10.663	10.661
**MEAN**	**32.567**	**32.565**	**32.561**	**63.558**	**63.556**	**63.554**	**51.433**	**51.431**	**51.428**	**18.239**	**18.237**	**18.234**
